# Improving Access to Addiction Recovery Care in Central Appalachia Through Organizational Collaboration

**DOI:** 10.13023/jah.0102.07

**Published:** 2019-07-06

**Authors:** Katy Stigers

**Keywords:** Appalachia, coalition, addiction recovery, employment, organizational collaboration, collective impact

## Abstract

Fahe, a Network of 50+ members throughout Appalachia based in Berea KY, has brought together a coalition to finance, build, and manage several addiction recovery care centers across Kentucky and West Virginia, increase access to employment, and deploy vouchers for supportive services.

Collective Impact (CI), an approach coined by Kania and Kramer, has been applied to urban settings to bring groups together around a shared outcome.^1^ Its value in addressing rural issues is uncertain. CI defines five necessary conditions: common agenda, shared measurement system, mutually reinforcing activities, continuous communication, and backbone support. Backbone organizations use dedicated staff, resources, and skills to convene and coordinate participating organizations.^2^ In Central Appalachia, a CI-like approach, facilitated by a backbone organization, is taking shape to reverse the ravages of drug addiction.

Fahe, a Network of 50+ members throughout Appalachia based in Berea KY, has brought together a coalition to finance, build, and manage several addiction recovery care centers across Kentucky and West Virginia, increase access to employment, and deploy vouchers for supportive services. Fahe supports partners to align resources that bring housing, employment, treatment, and recovery support to communities. As with other CI efforts, mutually reinforcing activities will increase the impact of this work. The social determinants of health and recovery are also incorporated in this effort. The approach includes supportive services, finance for housing, shared management, and adaptive reuse. The partners bring expertise in medicine and social work, administration of federal programs, and construction.

Supplying housing, services, and treatment is complex and expensive. Projects soon to be underway include:

- *Recovery, Hope, Opportunity, and Resiliency program* received $1.6 million through ARC POWER funding for The Fletcher Group of Lexington KY to partner with Kentucky Community and Technical College System, Fahe, Kentucky Housing Corporation, Recovery Kentucky, Operation Unite, and others to eventually serve 300 individuals. The project will ultimately leverage $24 million in private capital and up to $4.8 million in other funding. Sites in Kentucky and West Virginia are planned. Fahe will serve as construction finance manager.
- *DV8* Fahe received $1,000,000 from ARC to fund a Second-Chance Employment project for individuals working to recover from substance abuse addiction. Partnering with local employers in six coal-impacted communities in eastern Kentucky, Fahe will adapt the model of Lexington KY social enterprise DV8 to educate employers and support them and their new employee to build a successful on-ramp to a job that offers training and pays above average wages. The project will place 30 people in employment.
- *Access to Recovery Voucher Program* Fahe, in collaboration with the commonwealth of Kentucky, will administer $3.7 million awarded by Substance Abuse and Mental Health Services Administration (SAMHSA) to fund recovery supportive services. The extensive Network of Members and Partners, nonpartisan orientation, collaborative model, and administrative capacity positioned Fahe to function as a backbone organization in this effort.

One feature of these developments is that they are possible due to a high-functioning, high-capacity network of locally based organization. These organizations benefit from the communication and coordinating support of a backbone organization. Fahe’s Member Network has charged it with facilitating connections with new partners and seeking opportunities to meet the challenges they have identified at the local level. Because of the diverse expertise among partners, it is possible to address more than one facet of addiction recovery in a holistic, regional approach to treatment.

The need is critical. According to Centers for Disease Control and Prevention mortality data, residents of the Appalachian region were 61% more likely to die from a drug overdose than residents of the rest of the U.S. in 2016. In Mingo County WV, the overdose mortality rate is 98.7 per 100,000 of population aged 15–64. Counties in eastern Kentucky are also seriously affected. Several have overdose rates at the far extremes, such as Leslie County with an overdose rate of 86.1 per 100,000 of population aged 15–64. Research conducted on behalf of the Appalachian Regional Commission (ARC) found that Central Appalachians are dying from “diseases of despair” (suicide, alcoholism, poisoning) at rates far higher than other subregions—including a shocking 80% higher than in Southern Appalachia. For people in recovery, access to housing and supportive services can sustain long-term success. Due to the buy-in and cooperation of the partners, the projects listed above are not disparate, single-purpose efforts but aligned and directed work toward a common outcome for the people of Central Appalachia.
